# Affordable Care Act Medicaid expansion and access to primary-care based smoking cessation assistance among cancer survivors: an observational cohort study

**DOI:** 10.1186/s12913-022-07860-3

**Published:** 2022-04-12

**Authors:** Steffani R. Bailey, Robert Voss, Heather Angier, Nathalie Huguet, Miguel Marino, Steele H. Valenzuela, Katherine Chung-Bridges, Jennifer E. DeVoe

**Affiliations:** 1 Department of Family Medicine, Oregon Health & Science University, 3181 SW Sam Jackson Park Road, Portland, OR USA; 2 OCHIN, Inc, 1881 SW Naito Parkway, Portland, OR USA; 3 Division of Biostatistics, School of Public Health, Oregon Health & Science University – Portland State University, 3181 SW Sam Jackson Park Road, Portland, OR USA; 4 Health Choice Network, 9064 NW 13th Terrace, Miami, FL USA

**Keywords:** Cancer survivors, Affordable care act, Health insurance, Smoking, Tobacco treatment, Community health centers

## Abstract

**Background:**

Smoking among cancer survivors can increase the risk of cancer reoccurrence, reduce treatment effectiveness and decrease quality of life. Cancer survivors without health insurance have higher rates of smoking and decreased probability of quitting smoking than cancer survivors with health insurance. This study examines the associations of the Affordable Care Act (ACA) Medicaid insurance expansion with smoking cessation assistance and quitting smoking among cancer survivors seen in community health centers (CHCs).

**Methods:**

Using electronic health record data from 337 primary care community health centers in 12 states that expanded Medicaid eligibility and 273 CHCs in 8 states that did not expand, we identified adult cancer survivors with a smoking status indicating current smoking within 6 months prior to ACA expansion in 2014 and ≥ 1 visit with smoking status assessed within 24-months post-expansion. Using an observational cohort propensity score weighted approach and logistic generalized estimating equation regression, we compared odds of quitting smoking, having a cessation medication ordered, and having ≥6 visits within the post-expansion period among cancer survivors in Medicaid expansion versus non-expansion states.

**Results:**

Cancer survivors in expansion states had higher odds of having a smoking cessation medication order (adjusted odds ratio [aOR] = 2.54, 95%CI = 1.61-4.03) and higher odds of having ≥6 office visits than those in non-expansion states (aOR = 1.82, 95%CI = 1.22-2.73). Odds of quitting smoking did not differ significantly between patients in Medicaid expansion versus non-expansion states.

**Conclusions:**

The increased odds of having a smoking cessation medication order among cancer survivors seen in Medicaid expansion states compared with those seen in non-expansion states provides evidence of the importance of health insurance coverage in accessing evidence-based tobacco treatment within CHCs. Continued research is needed to understand why, despite increased odds of having a cessation medication prescribed, odds of quitting smoking were not significantly higher among cancer survivors in Medicaid expansion states compared to non-expansion states.

## Introduction

Approximately 30% of cancer deaths in the United States (US) are caused by tobacco use and smoking [1]. Smoking among cancer survivors can increase the risk of cancer reoccurrence, reduce treatment effectiveness and survival time, and decrease quality of life [2]. Conversely, quitting smoking after a cancer diagnosis is associated with greater response to cancer treatment and reduced risk of other health conditions (e.g., heart disease, chronic obstructive pulmonary disease, stroke) [3]. The 2020 US Surgeon General’s report on smoking cessation suggests that quitting smoking after a cancer diagnosis can significantly reduce all-cause mortality relative to continued smoking [4].

A study using data from the 2017 National Health Interview Survey (NHIS) found 13% of adult cancer survivors reported current smoking [5] and approximately 44% of cancer survivors who previously smoked quit after cancer diagnosis. Indeed, cancer survivors are less likely to currently smoke and are more likely to report former smoking than those with no history of cancer [6]. A 2000-2017 national US study found higher probability of reporting a smoking cessation event after cancer diagnosis among cancer survivors who are older, those diagnosed as having a smoking-related cancer (vs. non-smoking-related), individuals with an undergraduate degree or a postgraduate degree (vs. high school diploma or GED), and individuals with obesity. Individuals living below the federal poverty level (FPL) have a lower probability of reporting a smoking cessation event after cancer diagnosis than those living above the FPL [7].

Cancer survivors without health insurance have higher rates of smoking and decreased probability of quitting smoking than cancer survivors with insurance [8–10]. A study using the NHIS from 2008 through 2017 data found a decreasing trend in smoking rates for cancer survivors with private insurance (17% in 2008/2009 to 12% in 2016/2017). This same study reported strikingly higher rates of smoking among uninsured cancer survivors across this time period, with 43% reporting current smoking in both 2008/2009 and 2016/2017 [11]. An earlier study using data from the 2009 Behavioral Risk Factor Surveillance System survey (ages 18-64) reported similar findings, with 41% prevalence of smoking among cancer survivors who did not have health insurance compared to 20% among those with health insurance. Further, uninsured cancer survivors had 2-fold greater odds of not quitting smoking compared to those with health insurance [10]. Therefore, insurance coverage could play a role in increasing access to smoking cessation assistance and increased cessation among cancer survivors.

The Patient Protection and Affordable Care Act (ACA) mandate called for the expansion of Medicaid coverage (the US public health insurance program for people with low incomes) to all adults earning less than or equal to 138% of the federal poverty level (FPL). Following a Supreme Court ruling, states were allowed to choose whether or not to expand Medicaid. As of February 2022, 39 states had adopted the Medicaid expansion and 12 states had not [12].

The ACA mandate also required that insurers cover certain preventive services, including smoking cessation interventions, with no cost sharing for newly eligible Medicaid beneficiaries in states that expanded Medicaid [13]. This provided the opportunity to have evidence-based tobacco treatment included as a covered benefit for millions of adult smokers not eligible for Medicaid pre-expansion [14]. Findings are mixed from studies examining the impact of the ACA on smoking-related outcomes among populations not limited to cancer survivors [15–21]. We are not aware of any studies that assessed smoking cessation assistance and quitting smoking among cancer survivors after the implementation of the ACA.

Therefore, this retrospective observational cohort study sought to examine the odds of smoking cessation assistance and cessation among cancer survivors who were patients of community health centers (CHCs) in states that expanded Medicaid via the ACA compared to those in non-expansion states. CHC settings are important to study given that they provide primary care services, including smoking cessation services, to many uninsured or Medicaid-insured patients with higher rates of smoking than the general population [22, 23]. National quality performance data show that CHCs have high rates of assessing and treating tobacco use among their patients [24, 25]. We tested the hypotheses that cancer survivors in ACA expansion states who reported current smoking prior to the ACA expansion would have higher odds of smoking cessation, higher odds of having a cessation medication ordered, and increased primary care utilization post-ACA compared to those in non-expansion states.

## Methods

### Data sources

We used electronic health record (EHR) data from the Accelerating Data Value Across a National Community Health Center Network (ADVANCE) Clinical Research Network (CRN) [26], a member of PCORnet. The ADVANCE CRN’s data warehouse integrates outpatient EHR data from several data networks. This study used data from OCHIN (not an acronym) and Health Choice Network (HCN).

### Study setting and population

In this observational cohort study, we included 337 primary care CHCs across 12 states that expanded Medicaid eligibility to ≤138% of the FPL for all adults including those without dependent children as of 1/1/2014 (California, Hawaii, Massachusetts, Maryland, Minnesota, New Mexico, Nevada, Ohio, Oregon, Rhode Island, Washington, and Wisconsin) and 273 CHCs in 8 non-expansion states (Alaska, Florida, Indiana, Kansas, Missouri, Montana, North Carolina, and Texas). We included Wisconsin as an expansion state because it opened Medicaid coverage to adults earning up to 100% of the FPL (near the threshold of ≤138% of the FPL). We included Alaska, Indiana, and Montana as non-expansion states because they did not expand Medicaid until late in our post-ACA study period (expanded 9/1/2015, 2/1/2015, and 1/1/2016 respectively). Our designation of states as expansion vs. non-expansion are similar to other studies assessing the impact of the ACA on health care outcomes and utilization [6, 17, 27–29].

We assessed patients 12 months pre-ACA (1/1/2013-12/31/2013) and 24 months post-ACA Medicaid expansion (1/1/2014-12/31/2015). We identified cancer survivors through their medical histories, encounter diagnoses, and problem-list records up to the date of their last pre-ACA visit. We included cancer survivors aged 19-64 throughout the entire study period, who had ≥1 pre-ACA visit to a study CHC and whose last recorded tobacco use status during this time period indicated current smoking (e.g., current every day, current some-day, heavy), and who had ≥1 post-ACA visit with a documented smoking assessment. Based on the US National Cancer Institute’s definition, we consider a cancer survivor to be anyone alive who has ever had a cancer diagnosis regardless of where they are in the course of their disease [30]. We excluded pregnant women as rates of care utilization and cessation treatment recommendations differ for this subgroup.

### Variables

#### Primary outcomes

Outcomes included quitting smoking (≥1 report of quitting), provision of smoking cessation medication (≥1 prescription of a cessation medication) and utilization of primary care (≥6 vs. < 6 visits) in the 24-month post-period. The EHR presents a discrete data field for smoking status at each primary care encounter, which can be confirmed, updated, or not reviewed. Smoking cessation (‘quit’) at ≥1 visit during the post-period was coded as a binary yes/no variable. Using methods similar to prior EHR-based studies [31–35], a person was identified as ‘quit’ if there was at least one measurement documented in the post-period that indicated the patient’s status changed from one indicating current smoking to one indicating former smoking (regardless of whether the patient had a subsequent status that indicated a return to smoking). For smoking cessation medication provision, we extracted orders for bupropion, varenicline, and all nicotine replacement products from EHR medication orders. As a proxy for utilization of care, we extracted data on the number of post-period office visits per patient (≥6 vs. < 6 visits) based on previous studies [31, 36].

#### Independent variable

Our independent variable was Medicaid expansion status: patients from CHCs in states that expanded versus did not expand.

#### Patient characteristics

To describe patients living in expansion and non-expansion states and to develop weights to control for differences in expansion groups, we used the following, EHR-derived pre-ACA covariates: sex, age as of 1/1/2014, race/ethnicity (Hispanic, non-Hispanic white, Non-Hispanic black, Non-Hispanic other, Missing), household income as percent of FPL as of 1/1/2014, location of patient’s primary clinic (urban/rural), number of ambulatory visits in 2013, insurance status at visits in 2013 (all private insurance; all or some visits with public insurance; discontinuously insured; all uninsured), and the Charlson Comorbidity Index [37]. We excluded both cancer and depression diagnosis from the Charlson Comorbidity Index since all patients in the study were cancer survivors and we included depressive disorders as a separate variable in our model since bupropion can be used for both depressive disorders and as a smoking cessation aid. We excluded 19 patients who were missing clinic location data.

### Analysis

To analyze whether cancer survivors in expansion states who reported current smoking prior to the ACA expansion would have better smoking cessation outcomes than those in non-expansion states, we used a propensity score (PS) weighted approach.

#### Propensity score weighting

We calculated inverse probability of treatment weights (IPTW) to control for differences in pre-ACA patient-level characteristics between the expansion and non-expansion groups. In this approach, patients are weighted by the inverse of the probability of being sampled from the treatment (i.e., expansion) group. We first fit a logistic regression model for expansion status including all patient characteristics described above as covariates, to obtain each patient’s PS or probability of being in an expansion state. We then calculated stabilized IPTWs to create a pseudo-population close to our original sample size. To assess balance before and after weighting, we computed standardized differences, as they are not unduly influenced by sample sizes. We considered covariates with absolute standardized mean differences (ASMD) ≤0.1 in the weighted sample to be free of residual imbalance that would influence final models. We calculated effective sample size, which was the approximate number of observations under simple random sampling that would produce a variation equivalent to that of the IPTW sample.

#### Generalized estimating equation logistic regression

Using the PS-weighted sample, we computed adjusted odds ratios (aOR) and predicted marginal probabilities of quitting smoking, having a cessation medication order, or having ≥6 ambulatory visits in the post-period. We used generalized estimating equation (GEE) logistic regression models to account for clustering of patients within CHCs. To account for potential differences in data handling, we included EHR network (i.e. OCHIN vs. HCN) as the only covariate in our GEE models. All GEE models assumed an exchangeable correlation structure and applied a robust sandwich variance estimator to account for possible misspecification [38]. All analyses were conducted using SAS v9.3. The study was approved by the Oregon Health & Science University Institutional Review Board.

## Results

Table 1 displays the patient characteristics in Medicaid expansion and non-expansion states, before and after PS weighting. Our final sample included 476 cancer survivors from non-expansion states and 2441 from expansion states. The majority of our study sample were older (50-64 years of age), female, non-Hispanic white, publicly insured or uninsured, seen in urban clinics, and had multiple comorbidities. After IPTW, the characteristics of patients in expansion versus non-expansion states were well balanced. IPTW adjusted rates for quit status, smoking cessation medication orders, and ≥ 6 visits over 24 months in the overall study sample were 15.5, 28.5 and 59.2%, respectively.

**Table 1:** Patient characteristics as of 1/1/2014 (unless otherwise noted) among cancer survivors in Medicaid expansion and non-expansion states, before and after inverse probability of treatment weighting

Characteristic	Original Sample			Propensity-weighted Sample		
	Non-Expansion	Expansion		Non-Expansion	Expansion	
	(*n* = 476) %	(*n* = 2441) %	ASMD	(ESS = 471.32) %	(ESS = 2439.85) %	ASMD
Age group			*0.1068*			*0.0319*
18-39	9.2	11.8		11.7	11.4	
40-49	19.5	20.9		21.0	20.7	
50-64	71.2	67.4		67.3	67.9	
Sex			*0.0524*			*0.0743*
Female	64.5	67.0		70.2	66.8	
Male	35.5	33.0		29.8	33.2	
Race/ethnicity			*0.5032*			*0.0547*
Hispanic	9.7	5.7		6.3	6.4	
Missing	4.6	3.2		4.3	3.4	
Non-Hispanic Black	23.1	8.3		10.6	10.6	
Non-Hispanic Other	1.5	2.4		2.2	2.3	
Non-Hispanic white	61.1	80.5		76.7	77.4	
FPL %			*0.3538*			*0.0742*
≤ 138%	74.8	65.6		69.6	67.2	
> 138%	14.1	9.8		10.4	10.4	
missing	11.1	24.7		20.0	22.4	
Insurance status in 2013			*0.1108*			*0.0960*
Continuous Private	9.7	7.3		7.2	7.7	
Continuous or Some Public	53.2	56.0		58.0	55.5	
Continuous Uninsured	24.6	25.4		22.1	25.2	
Discontinuously Insured	12.6	11.3		12.7	11.6	
Ambulatory visits in 2013			*0.1888*			*0.0502*
1	10.9	14.7		12.9	14.0	
2-3	31.3	26.0		28.7	26.9	
4-6	32.6	28.9		30.2	29.5	
> 6	25.2	30.4		28.3	29.5	
Urban/rural clinic location			*0.236*			*0.0143*
Urban	80.3	88.7		88.0	87.6	
Rural	19.8	11.3		12.0	12.4	
Charlson Comorbidity Index			*0.0781*			*< 0.0001*
0 to 1	17.2	18.3		17.9	18.1	
2 to 3	22.7	19.8		20.1	20.3	
4 to5	29.2	31.4		31.3	30.9	
> 5	30.9	30.6		30.8	30.6	
Depressive disorder			*0.2187*			*0.0013*
Yes	76.7	66.9		68.6	68.5	
No	23.3	33.1		31.4	31.5	

Charlson Comorbidity Index value calculated without cancer or depression components

*ESS* effective sample size, *ASMD* absolute standardized mean difference, *FPL* federal poverty level

**Fig. 1:**
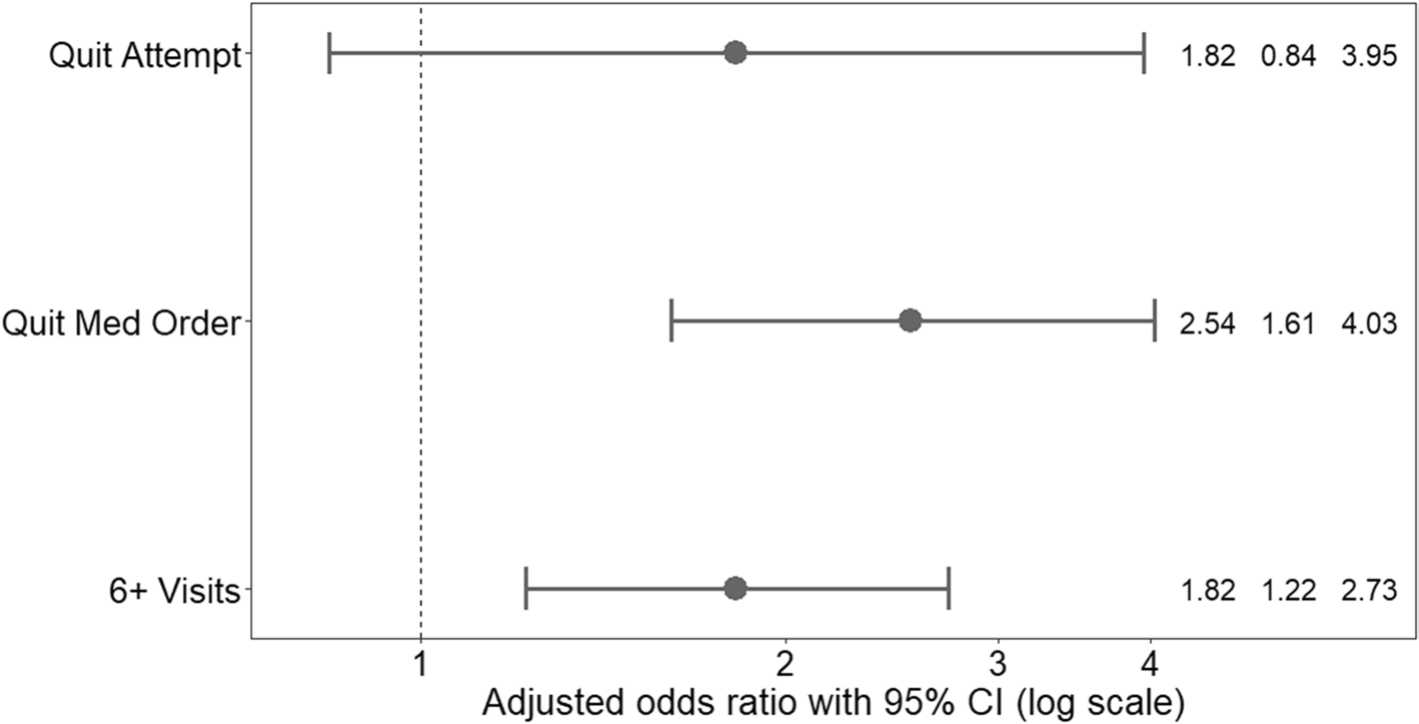
Adjusted odds ratios for quit status, smoking cessation medication ordered and ≥ 6 visits over 24 months comparing Medicaid expansion vs. non-expansion states (reference group) among cancer survivors. Note: OR: odds ratio; LCL: lower 95% confidence limit; UCL: upper 95% confidence limit. Results based on propensity score weighted logistic generalized estimating equation regression accounting for the following covariates: sex, age as of 1/1/2014, race/ethnicity, household income as percent of federal poverty level as of 1/1/2014, rurality/urbanity of patient’s primary clinic, number of ambulatory visits in 2013, insurance status in 2013, Charlson Comorbidity Index excluding cancer and depression as of 1/1/2014; depressive disorder as of 1/1/2014

Figure 1 presents aORs for our study outcomes as estimated by the GEE models. Table 2 also displays aORs, as well as predicted probabilities. Patients in expansion states had 2-fold greater odds of having a smoking cessation medication order (aOR = 2.54, 95% CI = 1.61-4.03), and 82% higher odds of having ≥6 visits than cancer survivors in non-expansion states (aOR = 1.82, 95%CI = 1.22-2.73). Patients in expansion states had a non-significant elevation in odds of quitting smoking compared to patients from non-expansion states (aOR = 1.82, 95%CI = 0.84-3.95).

**Table 2:** Predicted probabilities and adjusted odds ratios for quit status, smoking cessation medication ordered and ≥ 6 visits over 24 months comparing Medicaid expansion vs. non-expansion states (reference group) among cancer survivors

	Predicted Probability	95% CI		aOR	95% CI	
		Lower	Upper		Lower	Upper
**Quit Attempt**						
Expansion	0.145	0.126	0.167	1.824	0.843	3.948
Non-expansion	0.085	0.044	0.158	ref	ref	ref
**Quit Med Ordered**						
Expansion	0.308	0.2816	0.336	2.544	1.606	4.029
Non-expansion	0.149	0.1032	0.210	ref	ref	ref
**≥6 Office Visits**						
Expansion	0.611	0.5855	0.636	1.823	1.216	2.733
Non-expansion	0.463	0.3742	0.554	ref	ref	ref

*CI* confidence intervals, *aOR* adjusted odds ratios, *ref* reference group

## Discussion

The ACA expansion resulted in health insurance coverage for millions of US adults not previously Medicaid-eligible [14]. In the 24-months post-ACA expansion, cancer survivors from Medicaid expansion states had significantly higher odds of having a smoking cessation medication ordered and more primary care visits compared with those in non-expansion states. Research shows increased use of cessation treatments when out-of-pocket costs for these services are reduced or eliminated [39]; thus, removing cost-sharing among patients eligible via Medicaid expansion likely resulted in the higher rates of medication use. Insurance coverage also likely led to the increased visits among patients in Medicaid expansion states, which could have resulted in more opportunities to assess tobacco use and assist patients in quitting.

Despite these increased odds of smoking cessation medication orders among cancer survivors in expansion versus non-expansion states, we did not observe statistically significant differences in odds of quitting. These findings are in contrast to those of a previous study that found higher odds of quitting smoking among adult patients of CHCs in expansion states compared with those in non-expansion states; however, this prior study included the entire patient population and subanalyses for cancer survivors were not performed [20]. We postulate potential reasons for this finding. While the adjusted odds of quitting were higher for those in Medicaid expansion states (aOR = 1.82, 95%CI = 0.84-3.95), the lack of statistical significance could be due to low cell counts of those who quit, as shown in the wide confidence intervals.

It also is possible that current smoking cessation interventions, including pharmacotherapy, may not be as effective among cancer survivors compared to patients without a history of cancer. One meta-analysis of randomized controlled trials designed to promote smoking cessation among cancer survivors supports this conclusion; however, the quality of the included interventions was mixed and the authors caution in drawing firm conclusions based on the present evidence [40]. It also might be that cancer survivors need more intensive, tailored treatment to increase their odds of quitting smoking. Cancer survivors are more likely to experience poorer mental health than patients without cancer, including fear of cancer recurrence [41–43], which could impact smoking cessation outcomes [44, 45]. Given the association between smoking and negative affect, some cancer survivors might benefit from medications to treat the physical dependence, as well as more intensive or longer-term counseling to address the unique concerns and stressors of cancer survivors. A recent intervention study demonstrated that integrating evidence-based, sustained tobacco treatment (which included long-term telephone counseling) into the care of patients with cancer around the time of diagnosis can be effective [46]. Continued research is needed to identify and test smoking cessation interventions among cancer survivors, especially those in underserved communities.

The importance of CHCs for smoking cessation assistance among cancer survivors should also be highlighted. In the overall study sample, 30% of cancer survivors received smoking cessation medications from their CHC. The American College of Surgeons emphasizes that providing a high level of quality care to cancer survivors requires coordination of care among many medical disciplines, including primary care providers [47]. Much recent work has focused on increasing tobacco treatment in oncology settings, with cancer centers across the country receiving funds through the National Cancer Institute Cancer Moonshot Initiative to support this work [48]. While progress has been made in the oncology setting, reach has remained low [48]. Further, many patients return to primary care clinics rather than continue to receive ongoing care from cancer centers once treatment is complete [49–51]. Some consider, not only the diagnosis of cancer [3], but also the time period after active treatment to be a “teachable moment” (e.g., the time frame following a health event in which a patient is most conducive to behavioral changes) when cancer survivors might be focused on reducing the likelihood of cancer recurrence. This could lead to increased quit attempts [52]. Primary care clinics have the opportunity to address smoking cessation during this critical time. CHCs, the majority of which also have behavioral health services onsite [53], and insurance coverage are critical resources to ensure access to comprehensive treatment for smoking among this high-risk population.

### Limitations

We had follow-up smoking status for patients who had a return clinic visit, and therefore, cannot determine the quit status of patients who did not return for various reasons (e.g., transferred to another clinic, death). That said, a previous study found that about 80% of CHC patients with a chronic health problem return for ≥1 visit within a three-year period [54]. We also did not have detailed information on quit attempts, such as how long the patient remained quit or when the patient quit in relation to date of cancer diagnosis. Some evidence shows that quit attempts decline as the time since diagnosis increases [55]. We were unable to examine cessation by type of cancer as some counts were low or type of cancer was missing. Research suggests that smoking-related cancer survivors have higher current smoking prevalence [5, 56] and that odds of quitting smoking after a cancer diagnosis may vary by whether the person is a survivor of a smoking- or non-smoking-related cancer [5, 57]. We might not have captured use of nicotine replacement therapy that does not require a prescription. We also were unable to assess if bupropion was prescribed for smoking cessation or depression; however, all patients in the study sample reported current smoking and our models controlled for depressive disorders. We also only had access to medication orders, not receipt and/or use of the medications. We could not assess the impact of Medicaid expansion on the provision of cessation counseling among this population as these data were unavailable. While we were able to balance groups based on some known correlates of being uninsured and/or smoking (e.g., % of FPL, depressive disorders, race and ethnicity, clinic rurality, baseline assess to care), we were unable to control for all potential confounders, including state-level factors such as tobacco-related policies. Finally, due to use of diagnoses codes and problem lists to identify cancer survivors and only moderate agreement between CHC EHRs and cancer registries [49], some cancer survivors were likely not identified.

## Conclusions

Cancer survivors in ACA Medicaid expansion states had more than twice the odds of having a smoking cessation medication prescribed compared with those from non-expansion states, providing evidence of the importance of insurance coverage in accessing evidence-based tobacco treatment within the CHC setting. Our study findings support the need for continued efforts to ensure health insurance coverage for primary care-based tobacco treatment for socioeconomically disadvantaged cancer survivors. Further, research is needed to understand why, despite increased odds of having a cessation medication prescribed, odds of quitting were not significantly higher among cancer survivors in Medicaid expansion states. If our findings are replicated, interventions tailored to the specific needs of cancer survivors might be warranted.

## Data Availability

Raw data underlying this article were generated from multiple agencies and institutions; restrictions apply to the availability and re-release of data under cross-institution agreements.

## Abbreviations

ACA: Affordable Care Act
CHCs: Community Health Centers
NHIS: National Health Interview Survey
FPL: Federal poverty level
EHR: Electronic Health Record
ADVANCE: Accelerating Data Value Across a National Community Health Center Network
CRN: Clinical Research Network
HCN: Health Choice Network
IPTW: Inverse probability of treatment weights
ASMD: Absolute standardized mean differences
aOR: Adjusted odds ratios
GEE: Generalized estimating equation
